# Laser Actuation of Cantilevers for Picometre Amplitude Dynamic Force Microscopy

**DOI:** 10.1038/srep05567

**Published:** 2014-07-04

**Authors:** Drew R. Evans, Ponlawat Tayati, Hongjie An, Ping Koy Lam, Vincent S. J. Craig, Tim J. Senden

**Affiliations:** 1 Department of Applied Mathematics, Research School of Physics and Engineering, The Australian National University, Canberra, ACT, 0200, Australia; 2 Thin Film Coatings Group, Mawson Institute, University of South Australia, Mawson Lakes, SA 5095, Australia; 3 Department of Quantum Science, Research School of Physics and Engineering, The Australian National University, Canberra, ACT, 0200, Australia

## Abstract

As nanoscale and molecular devices become reality, the ability to probe materials on these scales is increasing in importance. To address this, we have developed a dynamic force microscopy technique where the flexure of the microcantilever is excited using an intensity modulated laser beam to achieve modulation on the picoscale. The flexure arises from thermally induced bending through differential expansion and the conservation of momentum when the photons are reflected and absorbed by the cantilever. In this study, we investigated the photothermal and photon pressure responses of monolithic and layered cantilevers using a modulated laser in air and immersed in water. The developed photon actuation technique is applied to the stretching of single polymer chains.

The Atomic Force Microscope (AFM) is widely used for high resolution imaging of surfaces, performing force analysis, surface characterization^1^,^2^, and manipulating molecular systems ranging from DNA^3^ and motor proteins to classical polymer chains^4^,^5^,^6^,^7^. The theoretical understanding of performing work on molecular systems is well established^8^,^9^,^10^, however, the experimental ability to perform nano-mechanical work on molecular systems has been problematic. The difficulty lies in the detection of changes in low energy regimes where the work performed on a system is comparable in magnitude to that of the work done by thermal fluctuations, due to Brownian motion. In this sense, the accuracy of AFM measurement at the molecular scale is said to be thermal fluctuation limited^11^,^12^,^13^. Minimizing the effect of thermal noise can be achieved either by averaging many measurements (although this has some inherent issues^14^) or by modulating the sensor such that the contribution of thermal noise at the modulation frequency is small in comparison to the total measureable signal. The latter principle is at the heart of dynamic force microscopy.

A key feature of dynamic AFM is the method used to produce the cantilever excitation. Usually, a piezo-electric actuator or magnetic particle are used to generate acoustic and magnetic excitation^15^,^16^, with the excitation method influencing the mode of vibration^17^. Recently it has been argued that piezoacoustic excitation of cantilevers can preclude accurate interpretation of data^18^. Optical excitation of AFM cantilevers has been achieved by modulating the intensity of a laser impinging on the cantilever both in air^19^,^20^,^21^ and in liquid^22^,^23^. This method produces a frequency response unaffected by spurious contributions of noise from mechanical coupling through liquids, thus providing an opportunity to explore details of hydrodynamic and fluid systems.

In this study, we use a modulated blue laser (wavelength: 405 nm) to excite an AFM cantilever. There are two ways to induce cantilever flexure via a laser: thermal heating, which leads to differential expansion and photon pressure. The latter was theoretically predicted by James Clerk Maxwell in the 1860's^24^, whereby light (or indeed any radiation) exerts a small force on the surface it impinges. The relationship between the power of the impinging radiation and the exerted force is given by Equation 1^25^. 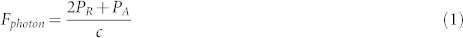
Where *F_Photon_* is the photon pressure force (N), *P_R_* is the fraction of photon power reflected (W) from the surface, *P_A_* is the fraction of absorbed photon power (W) and c is the speed of light (m/s). It must be noted here that the functional form of Equation 1 for a cantilever would require knowledge of reflectivity and absorptivity of the cantilever, which varies with the laser wavelength and optical geometry (angle of incidence).

## Methods

**Figure 1:**
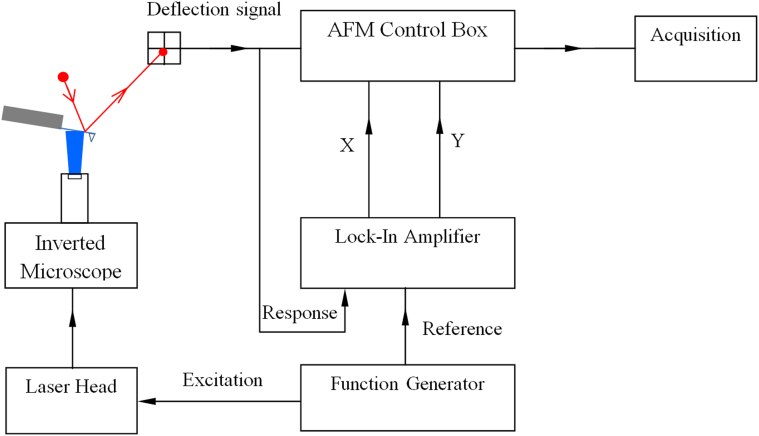
A schematic of the experimental configuration employed. The modulated laser beam is coupled to the AFM via an inverted optical microscope. In our arrangement the beam is not focussed to a point but rather floods the whole of the lower side of the cantilever. The influence of focussed beams on cantilever bending has been studied previously^22^,^23^. The deflection of the cantilever is measured using the conventional optical lever technique, reflecting from the upper side of the cantilever. The oscillatory response of the cantilever is isolated using a lock-in amplifier.

When a load is applied to a cantilever, it will deflect until a force balance is reached between the applied load and restoring force of the cantilever. Using the framework outlined in^26^, the photon pressure induced force per unit length of the cantilever, can be related to the measured deflection of the cantilever under a distributed load by Equation 2, 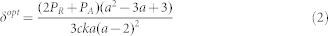
where, *δ^opt^* is the end load calibrated measured deflection from the optical lever (m) [ie the standard detection system used on many commercial instruments]; *a* is the normalised measurement position (*a = x/L* where *x* is the distance of measurement laser spot from the base of the cantilever and *L* is the length of the cantilever); and *k* is the spring constant of the cantilever (N/m). We use this equation to calculate the deflection measured using the optical lever technique for a given total laser power, and compare it to the observed deflection. For static measurements, δ^opt^ is a deflection of the cantilever caused by continuous laser illumination. To perform dynamic measurements, the cantilever is modulated and the response of the cantilever is observed at the actuated frequency. Given we are using a dynamic method, the amplitude response of the cantilever due to the photon pressure can be derived from Equation 2 as, 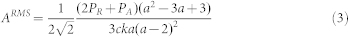
The amplitude response due to the photon pressure can be calculated for a cantilever with known spring constant, measurement position, and the reflected and absorbed photon power. Here we use a modulated laser beam, in the arrangement depicted in Figure 1, to fully illuminate the underside of an AFM cantilever, causing it to oscillate.

When light impinges on the AFM cantilever a portion of the light is absorbed by the cantilever, resulting in heating, the remainder is reflected. By conservation of momentum, there is a net transfer of momentum from the photons to the cantilever, causing the cantilever to deflect in response. To demonstrate this technique, an AFM (Research MFP-3D, Santa Barbara, CA) was modified to incorporate a modulated laser unit (Coherent Compass Laser, *λ* = 405 nm) where the modulation signal is produced from an arbitrary function generator (Hewlett Packard/Agilent HP33120A). The modulated laser beam was coupled to the AFM via an inverted microscope (Nikon Eclipse TE 2000-U). The dynamic deflection amplitude was adjusted by varying the total incident laser power using neutral density (ND) filters. The power output of the laser was measured using a power meter (Field master GS, Coherent). Cantilever deflection was detected using the inbuilt conventional optics of the Asylum AFM. The oscillatory component of the deflection signal was isolated using a lock-in-amplifier (Stanford Research Systems, SR 830 DSP), which was locked to the modulation frequency, as defined by the reference signal of the function generator.

A range of AFM cantilevers from different suppliers were used in this investigation with the following designations. Multi 75 G and Multi 75 are rectangular silicon cantilevers without coating and coated with aluminium respectively (Budget Sensors). Contact G are rectangular silicon cantilevers without a coating (Budget Sensors) CSG 10 are rectangular single crystal silicon cantilevers with a gold coating (NT-MDT). SNL-10 are V shaped silicon nitride cantilevers with a gold coating (Bruker). PNP-DB-00x are rectangular silicon nitride cantilevers without a coating. CSC-38 are rectangular silicon cantilevers with an aluminium coating (Mikromasch). Water used was purified using a MilliQ Gradient system. Silicon substrates were cleaned using a RF frequency water plasma (10 W 45 s). PNIPAM (M_W_ = 5035, Polydispersity Index (PI) = 2.1) was synthesised according to Zhou et al.^27^, precipitated twice from acetone/n-hexane and confirmed with ^13^C-NMR.

## Results and Discussion

The frequency response spectrum of a laser modulated AFM cantilever is complex. The photothermal amplitude is largest at low modulation frequencies as the cantilever is able to cool and heat on the time scale of the driving frequency and it oscillates between deflection positions corresponding to the warm and cool states. As the frequency is increased, the time available for cooling and heating is decreased and consequently the amplitude of oscillation decreases. At sufficiently high modulation frequencies the cantilever effectively remains at a constant temperature and oscillation due to the photothermal effect becomes negligible. In comparison the contribution due to photon pressure is not expected to show any frequency dependence. As the excitation frequency increases other bending modes can result in broadening of the resonance peak. Evidently the contribution due to photon pressure, will be most evident at frequencies sufficiently large that the photothermal oscillation is reduced but below frequencies where the resonance of the cantilever is evident. We call this frequency window the photon pressure regime.

**Figure 2:**
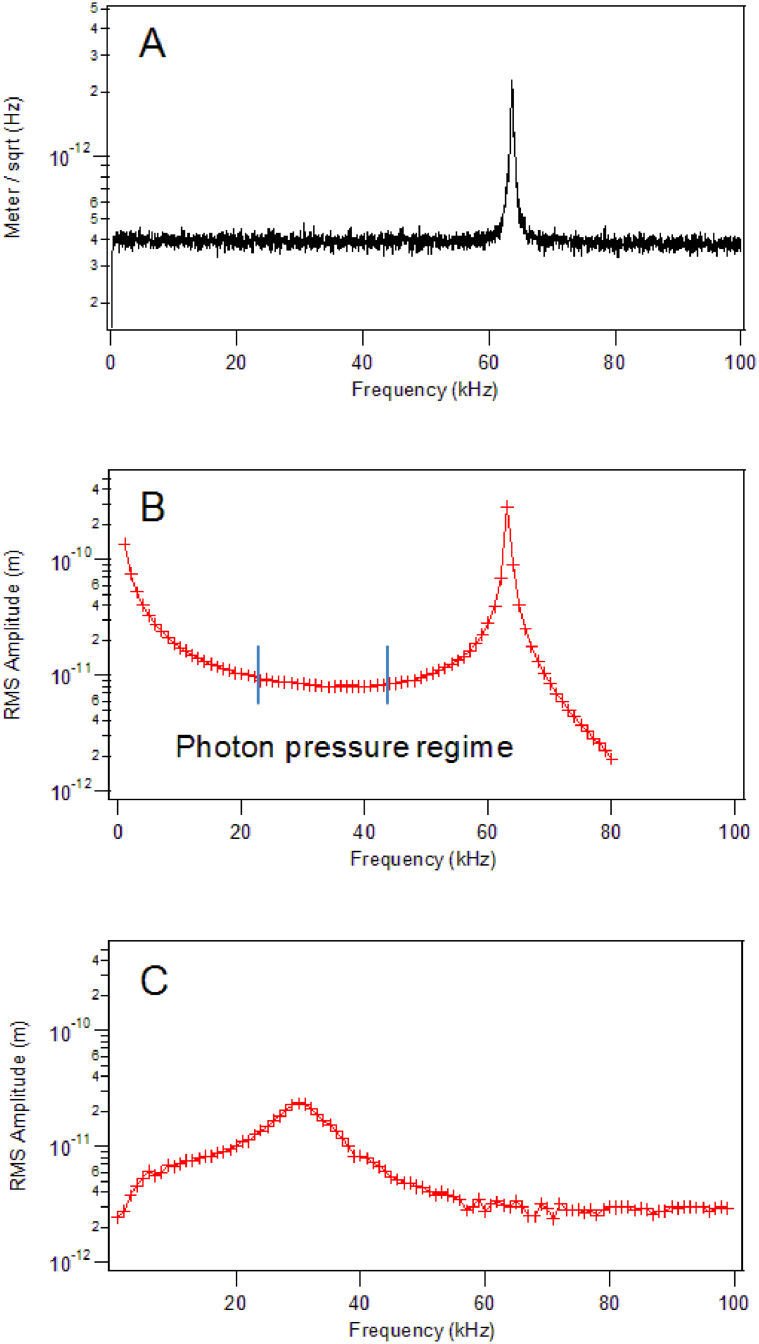
Amplitude versus frequency response of an aluminium coated silicon cantilever (Multi 75-Al). The thermally driven response spectrum (modulated laser is power off) is shown in Panel A. Laser actuated spectra obtained in air (Panel B) and water (Panel C) were obtained when the power of the impinging modulated laser was 7.5 μW.

The frequency response spectra of a Multi75-Al cantilever with and without laser excitation is shown in Figure 2. Without the additional excitation, the (thermally driven) frequency spectrum shows the fundamental resonance frequency of a cantilever to be 63.6 kHz with quality factor, Q = 155. The laser actuated spectrum yields approximately the same fundamental resonance frequency, however the peak is broadened. It is worth noting that the laser actuated spectrum does not show any additional spurious oscillation modes which would otherwise appear as artefacts in the spectrum, implying no other vibrational mode is excited, only the first flexural vibrational mode. The lower limit to the amplitude measurement is illustrated by obtaining a frequency response spectrum of the cantilever immersed in water. By immersing the cantilever in water, the resonance frequency is reduced (effective mass of the cantilever increases), the Q is reduced (increased damping) and it becomes possible to probe frequencies above resonance without exceeding the upper frequency limit of the lock-in amplifier used in our measurement (102 kHz). An example of this situation is shown in Figure 2C, where the measured RMS amplitude is several picometers at frequencies above the resonance for the first mode of vibration.

**Figure 3:**
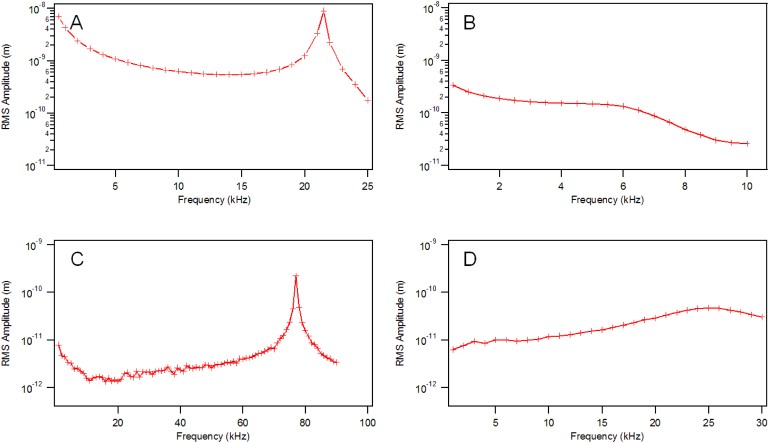
Amplitude response of a laser actuated gold coated silicon cantilever (CSG-10, 10 μW) (A) in air (B) in water; and an uncoated cantilever (Multi75-G, 7.5 μW) (C) in air and (D) in water.

**Figure 4:**
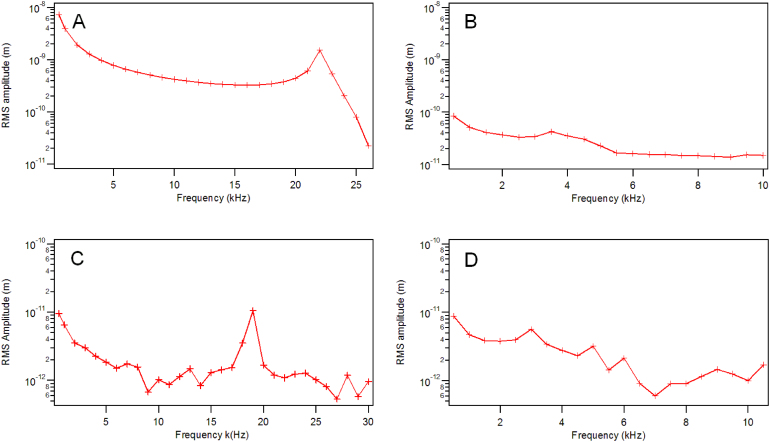
Amplitude response of a laser actuated gold coated silicon nitride cantilever (SNL-10, 16 μW) (A) in air (B) in water; and an uncoated cantilever (PNP-DB, 10 μW) (C) in air and (D) in water.

Both uncoated and metal-coated cantilevers could be excited in air and in water at room temperature (Figure 3 and Figure 4). The relative magnitude of the amplitude response for different types of cantilever actuated in air, with the same power level were quite different. The measured amplitude response from laser actuated cantilevers followed the trend: gold-coated cantilever > aluminium-coated cantilever > uncoated cantilever, for both silicon and silicon nitride cantilevers.

Considering the laser always strikes on the lower surface of all cantilevers examined, the reflection coefficient, was calculated according to Fresnel equations. The incident angle is typically ~10° due to the AFM instrument design, where a mounted cantilever is tilted relative to horizontal. Given the wavelength of light employed, 405 nm, the reflection coefficient for silicon and silicon nitride is 0.475 (refractive index 5.44^28^) and 0.125 (refractive index 2.07^29^) respectively. At thicknesses above 100 nm, silicon layers are opaque to light radiation with wavelengths of 250 nm to 500 nm. Therefore the transmission of light through the silicon cantilever can be neglected here^30^. The absorption coefficient of a typical silicon cantilever is 0.525. Since the absorption coefficient for silicon nitride is 10^5^ m^−1^^31^, the absorbed light intensity is 0.051 times the input intensity upon traversing a silicon nitride cantilever of 0.6 μm thickness, and the light reflected at the other side of the cantilever will contribute to the photon pressure through a second reflection. The intensity of the second reflection normalised by the incident radiation is 0.104. For simplicity we ignore subsequent reflections. The amplitude response from the photon pressure can then be calculated from Equation 3, giving an estimate of the amplitude from photon pressure (Table 1).

**Figure 5:**
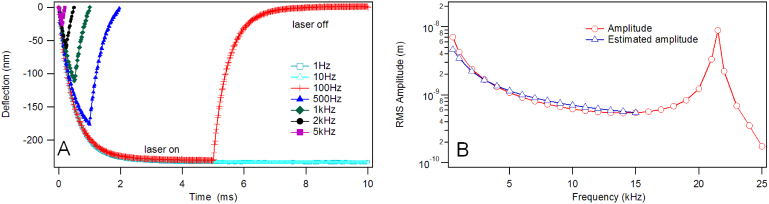
A) The bending of a coated cantilever (CSG-10, NTMDT) due to laser modulation with an incident power of 200 μW as a function of frequency and B) comparison of the measured and estimated thermal amplitude response for a coated cantilever (CSG-10, NTMDT), using an incident power of 10 μW.

In the photon pressure regime, the measured amplitudes of coated cantilevers are higher by 3 orders of magnitude than the estimated photon pressure amplitudes. For uncoated silicon cantilevers, the differences are 2 orders of magnitude. The amplitude response of uncoated silicon nitride cantilevers is closer to the estimated photon pressure amplitude, but still remains approximately 20 times higher. For coated cantilevers, the cantilever will bend due to difference in the thermal expansion coefficient of the coating and the substrate. The deflection can be calculated at a constant steady-state deflection for a uniformly distributed continuous laser using Equation 4^32^. 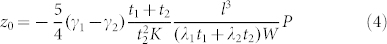
Where, z is the vertical deflection of cantilever, γ thermal expansion coefficient of layers, *t* the thickness of the layers, *l* the length, and *w* the width of the cantilever and *P* the absorbed power. The deflection of a coated cantilever actuated at different frequencies was plotted in Figure 5A. The response of the cantilever to heating effects can be fitted using an exponential, and thus Equation 4 can be modified into Equation 5 when a pulsed laser is employed. 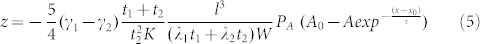
Where, τ is thermal relaxation time constant, and can be obtained by fitting an exponential to the deflection versus time curve. The thermal dynamic deflection amplitude can then be estimated (Figure 5B).

The bending of uncoated cantilevers can be induced due to a temperature gradient induced by the absorption of light. The temperature gradient and thermal expansion can be calculated according to Equation 6 and 7,^33^. 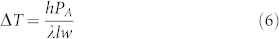

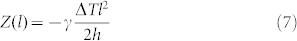
where *λ* is the thermal conductivity, *γ* thermal expansion coefficient, *h* the thickness of the cantilever, *l* its length, *w* its width, Δ*T* the temperature difference from the bottom to the top, and *Z(l)* is the thermal expansion caused by the temperature difference between the bottom and the top. For uncoated silicon cantilevers (Multi 75 G, *λ* = 149 W K^−1^ m^−1^, *γ* = 2.6 × 10^−6^ K^−1^, *l* = 225 μm, *w* = 28 μm, and *h* = 3 μm), the temperature difference between the bottom and the top of the cantilever is 16.8 μK for a total incident power of 10 μW and the resulting deflection at the end of the cantilever is 0.37 pm. Due to the low absorption coefficient of silicon nitride, γ = 10^5^ m^−1^, the absorbed power is *P_A_* = *P_in_*(1 − 0.125)(1 − e^−γz^) = 0.51 μW for a cantilever of 0.6 μm thickness and a total incident power of 10 μW. In comparison the calculated temperature difference from the bottom to the top of a 0.6 μm thick uncoated silicon nitride cantilever is only 1.2 μK (PNP-DB-00x, *λ* = 32 W K^−1^ m^−1^, *γ* = 3 × 10^−6^ K^−1^, *l* = 200 μm, *w* = 40 μm, and *h* = 0.6 μm). Assuming an emissivity of 1, the thermal expansion causes a deflection at the end of the cantilever of 0.12 pm. Whilst the manufacturers state that these cantilevers have no coating the silicon nitride will have a native oxide layer (~1 nm, thermal conductivity *λ* = 1.3 W K^−1^ m^−1^, *γ* = 5 × 10^−7^ K^−1^, young's moduli E = 70 GPa), therefore the cantilever will bend due to differential thermal expansion of this layer and the substrate. The bending due to differences in thermal expansion coefficients can be calculated using Equation (5). The calculated deflection of a silicon nitride cantilever due to thermal expansion with an oxide layer of thickness 1 nm at 10 kHz modulation of laser (power = 10 μW) is 0.1 pm. However, our data shows deflections several times the sum of the calculated thermal and photon pressure amplitudes. We note the silicon nitride cantilever is affixed to a substrate of Pyrex glass, having a different thermal expansion coefficient. It is possible that the stress in this joint is influenced by temperature. This may be the origin of the spurious oscillation of the cantilever.

In contrast to other modulation methods, the laser actuation technique has the ability to excite a cantilever at a frequency both above and well beyond the fundamental resonance frequency whilst maintaining a sub nanometre to nanometre oscillation amplitude. This enables time-resolved measurements to be performed as well as probing time-dependent dynamics of molecular systems.

We have applied our technique to time-dependent dynamics of molecular systems. The laser actuated AFM was employed to look at the dynamics of polymer chain stretching between an AFM tip and a silica substrate. The purpose of this example is to demonstrate the applicability of the technique. A detailed discussion of the dynamics of polymer chain extension forces is beyond the scope of this work^34^,^35^,^36^,^37^.

**Figure 6:**
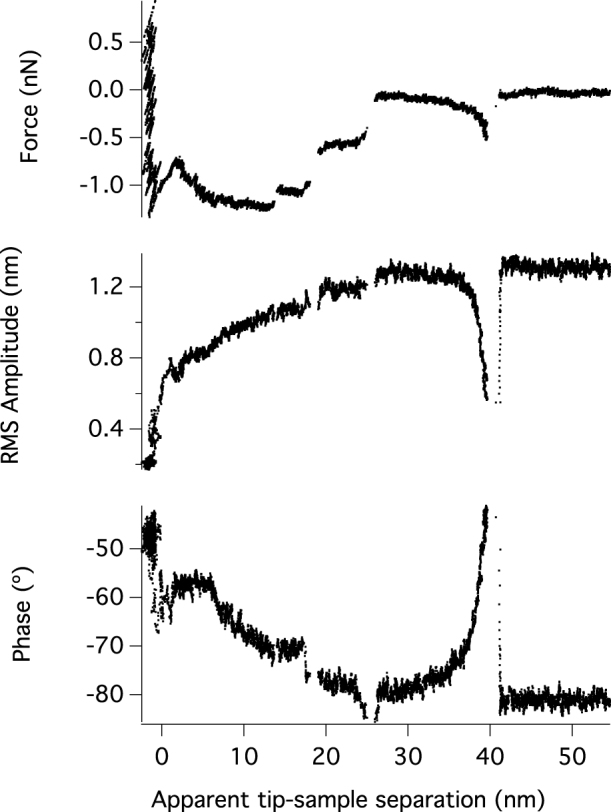
Measurements using a cantilever (CSC-38) driven by laser modulation at 6 kHz using with a free amplitude of 1200 pm, during the extension of a single Poly(N-Isopropylacrylamide) (PNIPAM) polymer chain from a silicon wafer in water. (A). The force versus separation measured in the conventional manner. (B). Amplitude of oscillation versus separation. (C). The relative phase response of the cantilever to the laser modulation.

**Figure 7:**
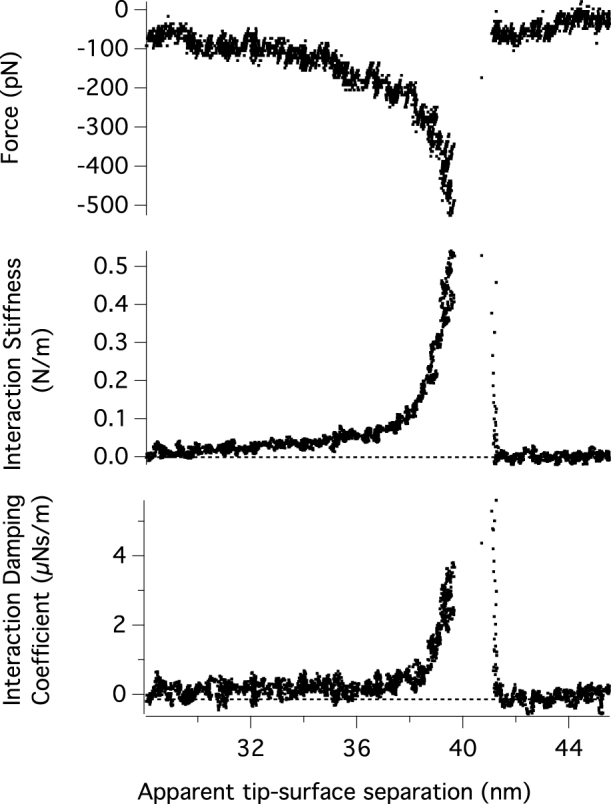
The force, interaction stiffness and damping coefficient of a photon pressure driven AFM cantilever during the extension of a single PNIPAM polymer chain. (A). A conventional force versus separation plot. (B). Interactions stiffness and (C) Interaction damping coefficient at 6 kHz using modulated laser excitation with a free amplitude of 1200 pm.

In our experiment, we measured the force required to stretch poly(N-isopropylacrylamide) (PNIPAM) chains which were adsorbed onto a glass slide in water (Figure 6). The tip has initially been brought into contact with the surface. Upon retraction, a polymer chain on some occasions forms a bridge between the two surfaces. The chain is then stretched as the tip is retracted further. As well as monitoring the conventional force-distance profile, we also monitored the oscillation amplitude and phase. We found the laser actuation technique was very sensitive to molecular events during single molecule extensions. This data can be used to calculate the interaction stiffness and damping coefficient using Equations (8) and (9). 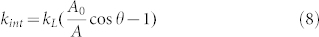

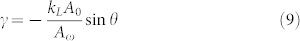
where *k_L_* is the cantilever stiffness, *A*_0_ and *A* are the free and interaction amplitudes respectively, *θ* is the phase (in radian) and *ω* is the angular frequency of cantilever oscillation^38^. 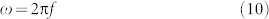
The results of an experiment where a single molecule is extended is shown in Figure 7. As the surfaces are separated a single molecule is stretched between the surfaces. This is revealed as an attractive force and increase in the interaction stiffness and damping of the system. At a tip-surface separation of 41.5 nm the bridging molecule is released and the measurements return to baseline values.

## Summary

We have demonstrated actuation of an AFM cantilever using a modulated laser and applied this to single molecule extensions. We found that the response of the cantilever is dominated by the photothermal effect, rather than photon pressure. This technique enables very small cantilever oscillation amplitudes to be employed over a wide frequency range. Armed with the ability to modulate a cantilever from sub-resonance frequencies up to and beyond the resonance frequency, time-dependent dynamics of molecular systems becomes accessible by AFM.

## Author Contributions

D.E., P.T., H.A., V.C. wrote the main manuscript. P.T., H.A. and D.E. prepared the figures. D.E., P.T., H.A., V.C., P.K.L. and T.S. reviewed the manuscript and contributed to the development of ideas.

## Figures and Tables

**Table 1:** Measured and estimated photon pressure amplitude of different cantilevers

Cantilever type	Spring constant (N/m)	Power (μW)	Measured Amplitude in the Photon pressure regime (pm)	Estimated Photon Pressure Amplitude (pm)
CSG-10	0.24	10	539	0.027
Multi 75-Al	1.21	7.5	7.9	0.004
Multi 75-G	1.81	7.5	1.33	0.003
Contact G	0.1	20	16.4	0.13
SNL-10	0.11	16	325	0.033
PNP-DB-00x	0.05	20	1.59	0.09
PNP-DB-00x	0.046	10	0.85	0.05
